# Practical Points

**Published:** 1895-05-25

**Authors:** 


### PRACTICAL POINTS.

*Hospital Floors.—Could anyone through your valuable paper advise me what to do for a corridor of an hospital that is boarded in blocks, and is splitting in various places—the boards are very soft—the more it is scrubbed the worse it gets? Our hospital is badly off for funds, so cannot lay out much expense. It is a new hospital, and I cannot understand the boards wearing like this.*

If this is a wood block floor laid on concrete the work must have been very badly done. For the rough wear of a corridor wood is unsuitable. The best thing to be done is to remove the wood and substitute artificial stone or terrazzo. If, however, this is quite out of the question, possibly the joints might be caulked (by a ship's joiner) with oakum and marine-glue. In any case, the floors should be treated with paraffin ironed in, hand-rubbed with oil and beeswax, washed as little as possible, but wiped over and dried when necessary. If the floor is boarded on joists, caulking and paraffining as above may help it; but soft wood is the worst material for hospital floors, and in the case in question, the more the floors are washed the more they will swell and shrink.

